# Polymorphisms of Insulin-Like Growth Factor 1 Pathway Genes and Breast Cancer Risk

**DOI:** 10.3389/fonc.2016.00136

**Published:** 2016-06-08

**Authors:** Joy Shi, Kristan J. Aronson, Anne Grundy, Lindsay C. Kobayashi, Igor Burstyn, Johanna M. Schuetz, Caroline A. Lohrisch, Sandip K. SenGupta, Agnes S. Lai, Angela Brooks-Wilson, John J. Spinelli, Harriet Richardson

**Affiliations:** ^1^Department of Public Health Sciences, Cancer Research Institute, Queen’s University, Kingston, ON, Canada; ^2^Individuals and Families, Alberta Cancer Prevention Legacy Fund, Alberta Health Services, Calgary, AB, Canada; ^3^Department of Epidemiology and Public Health, University College London, London, UK; ^4^Department of Environmental and Occupational Health, Drexel University, Philadelphia, PA, USA; ^5^Canada’s Michael Smith Genome Sciences Centre, British Columbia Cancer Agency, Vancouver, BC, Canada; ^6^Department of Medical Oncology, British Columbia Cancer Agency, Vancouver, BC, Canada; ^7^Department of Pathology and Molecular Medicine, Queen’s University, Kingston, ON, Canada; ^8^Department of Cancer Control Research, British Columbia Cancer Agency, Vancouver, BC, Canada; ^9^Department of Biomedical Physiology and Kinesiology, Simon Fraser University, Burnaby, BC, Canada; ^10^School of Population and Public Health, University of British Columbia, Vancouver, BC, Canada

**Keywords:** IGF, polymorphisms, breast cancer, breast cancer subtypes, interactions, case–control

## Abstract

Genetic variants of insulin-like growth factor 1 (IGF1) pathway genes have been shown to be associated with breast density and IGF1 levels and, therefore, may also influence breast cancer risk via pro-survival signaling cascades. The aim of this study was to investigate associations between IGF1 pathway single nucleotide polymorphisms (SNPs) and breast cancer risk among European and East Asian women, and potential interactions with menopausal status and breast tumor subtype. Stratified analyses of 1,037 cases and 1,050 controls from a population-based case–control study were conducted to assess associations with breast cancer for 22 SNPs across 5 IGF1 pathway genes in European and East Asian women. Odds ratios were calculated using logistic regression in additive genetic models. Polytomous logistic regression was used to assess heterogeneity by breast tumor subtype. Two SNPs of the *IGF1* gene (rs1019731 and rs12821878) were associated with breast cancer risk among European women. Four highly linked *IGF1* SNPs (rs2288378, rs17727841, rs7136446, and rs7956547) were modified by menopausal status among East Asian women only and associated with postmenopausal breast cancers. The association between rs2288378 and breast cancer risk was also modified by breast tumor subtype among East Asian women. Several *IGF1* polymorphisms were found to be associated with breast cancer risk and some of these associations were modified by menopausal status or breast tumor subtype. Such interactions should be considered when assessing the role of these variants in breast cancer etiology.

## Introduction

The insulin-like growth factor 1 (IGF1) signaling pathway has been implicated in normal cell growth, development, and differentiation in the mammary gland and other tissues (1, 2). Stimulation by growth hormone (GH) results in the production of IGF1, primarily in the liver. While IGF1 mediates its action through the insulin-like growth factor 1 receptor (IGF1R), its bioavailability is regulated by its binding proteins (IGFBPs). Over 90% of circulating IGF1 is bound by IGFBPs, with most being bound to insulin-like growth factor binding protein 3 (IGFBP3) (3). Downstream of IGF1R, signaling transmission by insulin receptor substrate 1 (IRS1) activates multiple intracellular signaling pathways that mediate the actions of IGF1. The two major pathways are the phosphoinositide 3-kinase (PI3K)/protein kinase B (Akt) pathway and the Ras/mitogen-activated protein kinase (MAPK) pathway (4).

Insulin-like growth factor 1 is suggested to play a role in the progression of many cancers, including prostate (5, 6), colorectal (7, 8), and breast (6, 9), as its pro-survival signaling pathways may encourage the proliferation of cancer cells (10). Epidemiological studies have previously demonstrated that elevated IGF1 concentrations are associated with increased risk of premenopausal breast cancer (11, 12), and current evidence also suggests similar associations between IGF1 concentrations and postmenopausal breast cancers (9). However, these associations may be restricted to estrogen receptor (ER)-positive tumors (9), mediated by synergistic growth effects from interactions between the estrogen and IGF1 signaling pathways when both are stimulated (13). Studies have also implicated IGFBP3 in breast cancer risk (6, 11, 12), although it is unclear whether this is a result of IGF-dependent or -independent functions (1).

Variants of the *IGF1* (14–16), *IGFBP3* (12, 14–17), *IGF1R* (18, 19), and *PI3KCB* (18) genes have been implicated in changes in circulating IGF1 and IGFBP3 levels, and may have systemic effects in the regulation of the IGF1 signaling pathway. However, the evidence for a relationship between variants in IGF-related genes and risk of breast cancer is less compelling. While some studies have identified associations with breast cancer risk for certain variants of IGF-related genes (15, 20–22), the Breast and Prostate Cancer Cohort Consortium (BPC3) genotyped 550 single nucleotide polymorphisms (SNPs) across 24 IGF1 pathway genes in a population of predominantly Caucasian postmenopausal women, but found no association with breast cancer risk (23). However, assessment of these associations in other ethnic groups and in premenopausal women, along with consideration of breast tumor subtypes, remains to be conducted.

We examined the associations of 22 polymorphisms across five IGF1 pathway genes (*IGF1*, *IGFBP3*, *IGF1R*, *IRS1*, and *PI3KCB*) with risk of breast cancer among women of European and East Asian descent. Potential interactions between IGF-related genes and menopausal status and breast tumor subtype were also assessed.

## Materials and Methods

### Study Population

To investigate the associations between IGF1 pathway genes and breast cancer risk, data from the Canadian Breast Cancer Study, previously known as the Molecular Epidemiology of Breast Cancer (MEBC) Study as described (24), were used. In brief, a case–control study was conducted in Vancouver, BC, and Kingston, ON, Canada. Incident cases from Vancouver and surrounding communities (*n* = 1,001) were recruited from the BC Cancer Registry and comprised women aged 40–80 newly diagnosed with *in situ* or invasive breast cancer with no previous history of cancer (except non-melanoma skin cancer). Age frequency-matched cancer-free controls (*n* = 1,014) were recruited from the Screening Mammography Program of BC. In Kingston, both cases (*n* = 131) and age frequency-matched controls (*n* = 163) were recruited from the Hotel Dieu Breast Assessment Program. All participants completed a questionnaire, either self-administered or by telephone interview, and provided a biological sample, either blood or saliva. Sufficient DNA for genotyping was extracted from blood and saliva samples for 92% of participants (*n* = 2,127) in the study. Pathologic data concerning tumor ER, progesterone receptor (PgR), and human epidermal growth factor receptor 2 (HER2) expression were obtained for most cases (*n* = 997, 96%) from both Vancouver and Kingston. Written informed consent was obtained from all participants and ethical approval for this study was obtained from the University of British Columbia – BC Cancer Agency Clinical Research Ethics Board and the Queen’s University Health Sciences Research Ethics Board.

### Data Collection

#### Study Questionnaire

The questionnaire completed by participants provided information regarding geographic ancestry; menopausal status; education; health, medical, and reproductive history; family history of cancer; lifestyle characteristics, including lifetime tobacco and alcohol consumption; and lifetime physical activity.

#### SNP Genotype Data

Genes involved in the synthesis (*IGF1*), bioavailability (*IGFBP3*), and downstream signaling (*IGF1R*, *IRS1*, *PI3KCB*) of IGF1 were selected. Based on suspected relationships with breast cancer risk or disease susceptibility from existing literature and using SNP tagging methods, a set of 28 SNPs were selected for genotyping. Selection of tagSNPs, using the CEU population from HapMap release 28 with a minimum minor allele frequency (MAF) of 0.10 and *r*^2^ threshold of 0.8, was conducted for three genes (*IGF1*, *IGFBP3*, and *PI3KCB*) using Tagger (25) in Haploview (26). Included in a 768-plex Illumina Golden Gate assay among SNPs related to other hypotheses were 11 SNPs of *IGF1*: rs6214, rs1549593 (15), rs17727841, rs2288378, rs7136446, rs2195239, rs7956547, rs1019731, rs12821878, rs1520220 (17), and rs2162679; 7 SNPs of *IGFBP3*: rs6670, rs2453839, rs3110697 (27), rs2471551 (15, 27), rs2132572 (15), rs10255707, and rs2854744 (15, 27, 28); 4 SNPs of *IGF1R*: rs951715 (29), rs2229765 (18, 29), rs8038415 (30), and rs9672965; 1 SNP of *IRS1*: rs1801278 (17); and 5 SNPs of *PI3KCB*: rs12493155, rs524164, rs10513055, rs361072 (17, 18), and rs693293. Genotyping was performed by the Genome Quebec/McGill University Innovation Centre in Montreal, Canada.

Quality control of genotype data was performed in Genome Studio v2011.1 (Illumina, San Diego, CA, USA), PLINK v1.07 (31), GRR (32), and Excel 2007 (Microsoft, Redmond, WA, USA), and has been previously described (24). Briefly, SNPs were excluded if they exhibited Gencall Scores <0.25, had GenTrain scores <0.4, exhibited poor clustering, were mono-allelic, showed genotype discrepancies in 126 pairs of replicate samples, had call rates <0.95, had unexpectedly low MAF among Caucasian controls in comparison to HapMap CEU data, or violated the Hardy–Weinberg equilibrium (*p* < 0.001) in European-ancestry controls. Relevant to this analysis, six SNPs were excluded following quality control: two by the genotyping center (rs1520220 and rs2854744), two for poor clustering (rs9672965 and rs693293), one for low call rate (rs10255707), and one for discrepancies in replicate samples (rs2162679). Therefore, final analyses included 22 IGF1 pathway SNPs.

Exclusion criteria for samples included call rate <0.95 (*n* = 14); unrelated samples having identical genotypes (*n* = 6); sex discrepancy, as identified by Y chromosome markers, indicating that the sample was male (*n* = 1); excess heterozygosity, specifically >3 SDs from the mean of other samples of the same ethnicity (*n* = 4); discrepancies between self-reported ethnicity and predicted ethnicity from genotype data (*n* = 5). If participants in the study were related, one was excluded (*n* = 9). Questionnaire data were used to verify that nine pairs of samples were from relatives. If the pairs were both cases, the individual with the later diagnosis date was excluded; if the pairs were both controls, the individual with the younger age was excluded. To compare self-reported ethnicity to predicted ethnicity, identity-by-state was calculated and multi-dimensional scaling plots (31) were created with HapMap samples selected from the CEU, CHB, CHD, JPT, YRI, and TSI populations. A total of 1,037 cases and 1,050 controls remained after quality control procedures.

### Assessment of Tumor Biomarkers

ER, PgR, and HER2 status of all invasive tumors was determined using immunohistochemistry (IHC), supplemented by fluorescence *in situ* hybridization (FISH) if the IHC results for HER2 were equivocal. Tumors were grouped according to biomarker status, regardless of intensity of expression. Four breast tumor subtypes were identified: ER+ and/or PgR+/HER2−, ER+ and/or PgR+/HER2+, ER−/PgR−/HER2+, and ER−/PgR−/HER2−.

### Statistical Analyses

Relationships between SNPs and breast cancer risk were investigated using unconditional logistic regression in an additive genetic model adjusted for age and center. All analyses were stratified by ethnicity and conducted in the two largest subgroups: Europeans and East Asians (Chinese, Japanese, Korean, and Filipino). The Benjamini–Hochberg adjustment (33) was used to control the FDR across all SNPs and provide corrected *p*-values. SNPs with a *q*-value (FDR adjusted *p*-value) <0.05 were considered statistically significant. Modification of these relationships by menopausal status was assessed by including an interaction term. Case–case polytomous logistic regression was used to assess heterogeneity of odds ratios between hormone receptor- and HER2-defined tumor subtypes. Assessment for these interactions was restricted to SNPs with *p*-value <0.1 for an association with breast cancer risk prior to false discovery rate (FDR) adjustment and MAF among controls >0.1 in each respective ethnic subgroup. All analyses were performed using SAS (Version 9.4, SAS Institute, Cary, NC, USA) and PLINK (31).

## Results

Characteristics of the study participants are presented in Table 1. In brief, approximately two-thirds of participants were women of European descent and one-quarter were of East Asian descent, although a greater proportion of cases were East Asian compared to controls. On average, breast cancer cases also had lower household income, were less likely to have completed postsecondary education, were older at their first mammogram, were more likely to have a primary family history of breast cancer and consumed less alcohol over their lifetime than controls.

**Table 1:** **Selected characteristics of study participants**.

	**Cases (*n* = 1037)** **Mean (SD)/*N*(%)**	**Controls (*n* = 1050)** **Mean (SD)/*N*(%)**
Age	57.0 (10.3)	56.5 (10.1)
Body mass index (kg/m^2^)	25.6 (5.4)	25.0 (4.7)
Ethnicity		
European	641 (61.8%)	806 (76.8%)
East Asian	305 (29.4%)	168 (16.0%)
South Asian	31 (3.0%)	31 (2.9%)
Mixed/other	60 (5.8%)	45 (4.3%)
Household income		
<$30,000	181 (17.5%)	104 (9.9%)
$30,000–$59,999	255 (24.6%)	230 (21.9%)
$60,000–$99,999	229 (22.1%)	262 (25.0%)
>$100,000	221 (21.3%)	310 (29.5%)
Not stated	151 (14.6%)	144 (13.7%)
Education		
High school or less	353 (34.3%)	259 (24.7%)
College/trade certificate	312 (30.3%)	310 (29.6%)
Undergraduate degree	244 (23.7%)	271 (25.9%)
Graduate/professional degree	121 (11.7%)	207 (19.8%)
Menopausal status		
Premenopausal	351 (33.9%)	391 (37.2%)
Postmenopausal	684 (66.1%)	659 (62.8%)
Reproductive history		
Age at menarche (years)	12.9 (1.6)	12.8 (1.5)
Ever been pregnant	856 (82.8%)	825 (78.7%)
Age at first birth (years)	27.8 (5.4)	27.7 (5.3)
Number of pregnancies	2.3 (1.7)	2.2 (1.7)
Age at first mammogram (years)	44.5 (8.6)	42.7 (7.4)
Family history of breast cancer	204 (19.7%)	145 (13.8%)
Current smoker	66 (6.4%)	62 (5.9%)
Pack-years smoking	5.7 (12.3)	5.1 (10.9)
Lifetime alcohol consumption (#drinks/week)		
Teen	1.1 (3.7)	1.6 (4.0)
20s	2.6 (5.5)	3.9 (8.1)
30s	2.9 (6.1)	3.5 (5.4)
40s	3.2 (6.1)	3.7 (5.6)
50s	2.8 (4.9)	3.8 (6.2)
Last 2 years	2.1 (4.5)	2.9 (5.0)
Moderate-to-vigorous physical activity (MET-hours/week)		
Leisure time	14.5 (19.0)	23.0 (32.3)
Household	39.7 (58.5)	48.9 (83.1)
Occupational	54.4 (76.8)	45.4 (66.4)
Medication use		
Oral contraceptive	570 (55.0%)	695 (66.2%)
Hormone replacement therapy	327 (31.7%)	351 (33.5%)

Relationships between SNPs and breast cancer risk among European and East Asian women are presented in Table 2. In the European group, the minor A-allele of three *IGF1* SNPs (rs1549593, rs1019731, and rs12821878) was associated with decreased risk of breast cancer, but only two of these SNPs passed adjustment for the FDR: rs1019731 (OR = 0.67, 95% CI: 0.53–0.84) and rs12821878 (OR = 0.73, 95% CI: 0.61–0.88). Among East Asians, no SNP was associated with breast cancer risk even prior to FDR adjustment.

**Table 2:** **Minor allele frequencies (MAF) and age- and center-adjusted odds ratios for the associations between SNPs in insulin-like growth factor signaling genes and breast cancer risk**.

Gene	SNP	Location	Major allele	Minor allele	European (641 cases, 806 controls)				East Asian (305 cases, 168 controls)			
					MAF (controls)	Odds ratio	*p*-value	*q*-value^a^	MAF (controls)	Odds ratio	*p*-value	*q*-value^a^
*IGF1*	rs6214	3′UTR	G	A	0.43	0.90 (0.77, 1.04)	0.16	0.47	0.49	0.83 (0.63, 1.09)	0.17	0.44
*IGF1*	rs1549593	Intron	C	A	0.16	0.76 (0.61, 0.95)	0.01	0.11	0.02	0.72 (0.25, 2.13)	0.56	0.68
*IGF1*	rs17727841	Intron	C	G	0.19	1.00 (0.82, 1.21)	0.96	0.99	0.19	0.73 (0.51, 1.03)	0.07	0.39
*IGF1*	rs2288378	Intron	G	A	0.25	1.06 (0.89, 1.26)	0.52	0.82	0.19	0.71 (0.50, 1.01)	0.06	0.39
*IGF1*	rs7136446	Intron	A	G	0.42	1.09 (0.93, 1.26)	0.29	0.61	0.19	0.71 (0.50, 1.01)	0.06	0.39
*IGF1*	rs2195239	Intron	G	C	0.25	0.98 (0.83, 1.17)	0.84	0.98	0.46	0.94 (0.72, 1.23)	0.65	0.75
*IGF1*	rs7956547	Intron	A	G	0.27	0.97 (0.82, 1.15)	0.71	0.89	0.18	0.71 (0.50, 1.02)	0.07	0.39
*IGF1*	rs1019731^b^	Intron	C	A	0.15	**0.67 (0.53, 0.84)**	<0.01	**0.01**	0.00	N/A	N/A	N/A
*IGF1*	rs12821878^c^	Intron	G	A	0.24	**0.73 (0.61, 0.88)**	<0.01	**0.01**	0.04	1.25 (0.63, 2.49)	0.53	0.68
*IGFBP3*	rs6670	3′UTR	A	T	0.23	0.95 (0.80, 1.14)	0.57	0.84	0.01	2.41 (0.82, 7.06)	0.11	0.40
*IGFBP3*	rs2453839	Intron	A	G	0.19	1.04 (0.87, 1.25)	0.66	0.89	0.24	0.83 (0.60, 1.14)	0.24	0.49
*IGFBP3*	rs3110697	Intron	G	A	0.43	0.93 (0.80, 1.08)	0.36	0.66	0.29	0.86 (0.64, 1.15)	0.30	0.55
*IGFBP3*	rs2471551	Intron	G	C	0.22	0.90 (0.75, 1.08)	0.26	0.61	0.02	1.77 (0.74, 4.24)	0.20	0.44
*IGFBP3*	rs2132572	Promoter region	G	A	0.21	1.00 (0.84, 1.20)	0.99	0.99	0.25	0.81 (0.59, 1.11)	0.18	0.44
*IGF1R*	rs951715	Intron	A	G	0.34	1.14 (0.98, 1.33)	0.08	0.36	0.50	0.89 (0.68, 1.16)	0.38	0.59
*IGF1R*	rs2229765	Exon^d^	G	A	0.44	1.03 (0.89, 1.19)	0.73	0.89	0.33	0.99 (0.74, 1.32)	0.95	0.99
*IGF1R*	rs8038415	Intron	A	G	0.49	1.00 (0.86, 1.15)	0.95	0.99	0.46	1.22 (0.93, 1.60)	0.15	0.44
*IRS1*	rs1801278	Exon^e^	G	A	0.06	1.17 (0.87, 1.57)	0.31	0.61	0.02	0.39 (0.13, 1.16)	0.09	0.39
*PI3KCB*	rs12493155	Intron	G	A	0.45	1.07 (0.92, 1.23)	0.40	0.67	0.48	0.88 (0.68, 1.15)	0.35	0.59
*PI3KCB*	rs524164	Intron	G	A	0.47	0.90 (0.78, 1.04)	0.16	0.47	0.02	0.70 (0.26, 1.92)	0.49	0.68
*PI3KCB*	rs10513055	Intron	A	C	0.23	0.85 (0.71, 1.01)	0.07	0.36	<0.01	0.58 (0.04, 9.37)	0.70	0.77
*PI3KCB*	rs361072	Intron	A	G	0.47	0.90 (0.78, 1.05)	0.17	0.47	0.02	0.70 (0.26, 1.92)	0.49	0.68

*SNPs with a *q*-value <0.05 are presented in bold*.

*^a^False discovery rate-adjusted *p*-value*.

*^b^Genotype frequencies of *n*_CC_ = 509, *n*_AC_ = 125, and *n*_AA_ = 7 among European cases; *n*_CC_ = 578, *n*_AC_ = 210, and *n*_AA_ = 18 among European controls*.

*^c^Genotype frequencies of *n*_GG_ = 421, *n*_AG_ = 195, and *n*_AA_ = 25 among European cases; *n*_GG_ = 461, *n*_AG_ = 301, and *n*_AA_ = 44 among European controls*.

*^d^Synonymous mutation*.

*^e^Gly/Arg substitution*.

Analyses further stratified by menopausal status are presented in Table 3. Among European women, no effect modification was detected. However, there was suggested modification by menopausal status for the rs1801278 polymorphism in the *IRS1* gene (interaction *p*-value, *p*_int_ = 0.06), which appeared to decrease the risk of premenopausal, while increasing the risk of postmenopausal breast cancers. Among East Asian women, modification of genetic effect by menopausal status was apparent for four *IGF1* SNPs: rs17727841, rs2288378, rs7136446, and rs7956547 (*p*_int_ < 0.01 for all), although these SNPs were in high linkage disequilibrium (LD) (*r*^2^ = 0.86–0.98). The minor alleles of these SNPs were associated with decreased risk of breast cancer among postmenopausal women.

**Table 3:** **Selected interactions of menopausal status and IGF1 pathway SNPs on breast cancer risk among European and East Asian women**.

Gene	SNP	European OR (95% CI)			East Asian OR (95% CI)	
		Premenopausal (189 cases, 275 controls)	Postmenopausal (451 cases, 531 controls)	*p*_int_^a^	Premenopausal (127 cases, 79 controls)	Postmenopausal (178 cases, 89 controls)	*p*_int_^a^
*IGF1*	rs6214	1.01 (0.77, 1.31)	0.85 (0.70, 1.02)	0.32	0.97 (0.65, 1.45)	0.68 (0.46, 0.99)	0.14
*IGF1*	rs1549593	0.95 (0.65, 1.40)	0.69 (0.53, 0.89)	0.17	0.83 (0.18, 3.81)	0.66 (0.14, 3.03)	0.84
*IGF1*	rs17727841^b^	0.97 (0.67, 1.41)	1.00 (0.80, 1.26)	0.89	**1.31 (0.76, 2.26)**	**0.44 (0.27, 0.72)**	**<0.01**
*IGF1*	rs2288378^c^	0.91 (0.65, 1.26)	1.12 (0.91, 1.38)	0.30	**1.31 (0.76, 2.26)**	**0.43 (0.26, 0.69)**	**<0.01**
*IGF1*	rs7136446^d^	0.90 (0.69, 1.19)	1.17 (0.97, 1.41)	0.13	**1.25 (0.73, 2.13)**	**0.45 (0.28, 0.72)**	**<0.01**
*IGF1*	rs2195239	1.07 (0.77, 1.48)	0.95 (0.77, 1.17)	0.55	1.00 (0.66, 1.53)	0.89 (0.62, 1.27)	0.66
*IGF1*	rs7956547^e^	0.97 (0.71, 1.33)	0.97 (0.79, 1.19)	0.99	**1.25 (0.72, 2.17)**	**0.45 (0.28, 0.73)**	**<0.01**
*IGF1*	rs1019731	0.66 (0.44, 1.00)	0.67 (0.52, 0.88)	0.93	N/A	N/A	N/A
*IGF1*	rs12821878	0.66 (0.48, 0.92)	0.77 (0.62, 0.96)	0.45	1.24 (0.47, 3.27)	1.28 (0.48, 3.41)	0.97
*IGFBP3*	rs6670	0.80 (0.58, 1.11)	1.02 (0.82, 1.27)	0.20	N/A	1.40 (0.43, 4.52)	N/A
*IGFBP3*	rs2453839	0.98 (0.71, 1.36)	1.08 (0.86, 1.35)	0.65	0.85 (0.55, 1.33)	0.82 (0.52, 1.29)	0.89
*IGFBP3*	rs3110697	0.96 (0.73, 1.27)	0.92 (0.77, 1.10)	0.79	0.93 (0.60, 1.44)	0.79 (0.52, 1.19)	0.58
*IGFBP3*	rs2471551	0.83 (0.60, 1.15)	0.94 (0.75, 1.17)	0.52	7.42 (0.94, 58.67)	0.91 (0.33, 2.56)	0.08
*IGFBP3*	rs2132572	1.14 (0.81, 1.59)	0.95 (0.77, 1.17)	0.36	0.79 (0.51, 1.23)	0.82 (0.52, 1.30)	0.91
*IGF1R*	rs951715	1.34 (1.03, 1.75)	1.05 (0.87, 1.26)	0.13	0.83 (0.57, 1.21)	0.97 (0.67, 1.40)	0.56
*IGF1R*	rs2229765	1.10 (0.84, 1.43)	1.00 (0.84, 1.19)	0.60	0.82 (0.54, 1.23)	1.22 (0.81, 1.84)	0.17
*IGF1R*	rs8038415	0.96 (0.74, 1.24)	0.98 (0.82, 1.17)	0.70	1.00 (0.68, 1.48)	1.48 (1.01, 2.17)	0.15
*IRS1*	rs1801278	0.77 (0.45, 1.31)	1.44 (1.00, 2.08)	0.06	0.31 (0.03, 3.43)	0.40 (0.12, 1.34)	0.85
*PI3KCB*	rs12493155	1.13 (0.87, 1.46)	1.04 (0.87, 1.24)	0.61	0.92 (0.61, 1.38)	0.85 (0.60, 1.20)	0.77
*PI3KCB*	rs524164	0.87 (0.68, 1.13)	0.92 (0.77, 1.10)	0.75	0.23 (0.04, 1.23)	1.78 (0.36, 8.75)	0.09
*PI3KCB*	rs10513055	0.82 (0.59, 1.13)	0.87 (0.70, 1.08)	0.76	N/A	N/A	N/A
*PI3KCB*	rs361072	0.87 (0.67, 1.12)	0.92 (0.77, 1.10)	0.70	0.23 (0.04, 1.23)	1.78 (0.36, 8.75)	0.09

*SNPs with a *p*-value for interaction by menopausal status are presented in bold*.

*^a^*p*-value for interaction by menopausal status*.

*^b^Genotype frequencies of *n*_CC_ = 86, *n*_CG_ = 37, and *n*_GG_ = 4 among premenopausal East Asian cases; *n*_CC_ = 60, *n*_CG_ = 16, and *n*_GG_ = 3 among premenopausal East Asian controls; *n*_CC_ = 141, *n*_CG_ = 33, and *n*_GG_ = 4 among postmenopausal East Asian cases; *n*_CC_ = 52, *n*_CG_ = 33, and *n*_GG_ = 4 among postmenopausal East Asian controls*.

*^c^Genotype frequencies of *n*_GG_ = 86, *n*_AG_ = 37, and *n*_AA_ = 4 among premenopausal East Asian cases; *n*_GG_ = 60, *n*_AG_ = 16, and *n*_AA_ = 3 among premenopausal East Asian controls; *n*_GG_ = 141, *n*_AG_ = 33, and *n*_AA_ = 4 among postmenopausal East Asian cases; *n*_GG_ = 51, *n*_AG_ = 34, and *n*_AA_ = 4 among postmenopausal East Asian controls*.

*^d^Genotype frequencies of *n*_AA_ = 86, *n*_AG_ = 37, and *n*_GG_ = 4 among premenopausal East Asian cases; *n*_AA_ = 59, *n*_AG_ = 17, and *n*_GG_ = 3 among premenopausal East Asian controls; *n*_AA_ = 139, *n*_AG_ = 35, and *n*_GG_ = 4 among postmenopausal East Asian cases; *n*_AA_ = 51, *n*_AG_ = 34, and *n*_GG_ = 4 among postmenopausal East Asian controls*.

*^e^Genotype frequencies of *n*_AA_ = 87, *n*_AG_ = 37, and *n*_GG_ = 3 among premenopausal East Asian cases; *n*_AA_ = 60, *n*_AG_ = 16, and *n*_GG_ = 3 among premenopausal East Asian controls; *n*_AA_ = 141, *n*_AG_ = 33, and *n*_GG_ = 4 among postmenopausal East Asian cases; *n*_AA_ = 52, *n*_AG_ = 34, and *n*_GG_ = 3 among postmenopausal East Asian controls*.

Lastly, heterogeneity in odds ratios between breast tumor subtypes for select IGF1 pathway SNPs among European and East Asian women are presented in Table 4. In European women, heterogeneity between tumor subtypes was not observed for any of the five SNPs that were examined. In East Asian women, tumor heterogeneity was observed for rs2288378 (p_TH_ = 0.04), in which a decreased risk of ER+ and/or PgR+/HER2− tumors, but not other tumor subtypes, was noted.

**Table 4:** **Heterogeneity in odds ratios between breast tumour subtypes for select IGF1 pathway SNPs among European and East Asian women**.

Ethnicity	Gene	SNP	Genotype	Tumor subtype								p_TH_^a^
				ER+ and/or PgR+/HER2+		ER+ and/or PgR+/HER2−		ER−/PgR−/HER2+		ER−/PgR−/HER2−		
				*n*	OR (95% CI)	*n*	OR (95% CI)	*n*	OR (95% CI)	*n*	OR (95% CI)	
European	*IGF1*	rs1549593	CC	56	0.82 (0.50, 1.36)	307	0.74 (0.57, 0.96)	25	0.64 (0.29, 1.44)	84	0.76 (0.50, 1.17)	0.96
			AC	16		93		7		26		
			AA	2		3		0		1		
European	*IGF1*	rs1019731	CC	62	0.49 (0.26, 0.89)	320	0.65 (0.50, 0.85)	25	0.67 (0.30, 1.50)	87	0.76 (0.50, 1.17)	0.68
			AC	12		82		7		21		
			AA	0		2		0		3		
European	*IGF1*	rs12821878	GG	52	0.61 (0.39, 0.96)	270	0.70 (0.57, 0.87)	20	0.71 (0.38, 1.35)	70	0.81 (0.57, 1.15)	0.79
			AG	20		118		12		36		
			AA	2		16		0		5		
European	*IGF1R*	rs951715	AA	22	1.60 (1.14, 2.24)	167	1.14 (0.96, 1.36)	12	1.14 (0.69, 1.91)	46	0.98 (0.73, 1.31)	0.16
			AG	36		172		16		55		
			GG	16		65		4		10		
European	*PI3KCB*	rs10513055	AA	49	0.74 (0.48, 1.15)	260	0.81 (0.66, 1.00)	16	1.11 (0.62, 1.99)	64	1.00 (0.71, 1.39)	0.45
			AC	23		130		16		43		
			CC	2		14		0		4		
East Asian	*IGF1*	**rs2288378**	**GG**	**24**	**0.86 (0.44, 1.66)**	**148**	**0.54 (0.35, 0.82)**	**7**	**1.24 (0.49, 3.17)**	**37**	**1.15 (0.68, 1.93)**	**0.04**
			**AG**	**12**		**37**		**6**		**11**		
			**AA**	**0**		**2**		**0**		**6**		

*SNPs with a *p*-value for tumour heterogeneity are presented in bold*.

*^a^*p*-value for tumor heterogeneity in odds ratio by breast tumor subtype*.

## Discussion

In our analyses of European and East Asian women, we observed associations between select *IGF1* SNPs and breast cancer risk in both European and East Asian women. In Europeans, the minor alleles of two *IGF1* SNPs (rs1019731 and rs12821878) were associated with reduced breast cancer risk. In East Asians, the association between four *IGF1* SNPs (rs17727841, rs2288378, rs7136446, and rs7956547) in high LD and breast cancer risk was modified by menopausal status and the minor alleles of these SNPs were associated with decreased risk of postmenopausal breast cancer. Lastly, heterogeneity by tumor subtype for rs2288378 was observed in East Asian women.

An association between SNPs of the IGF1 pathway and breast cancer risk has been hypothesized due to the role of circulating IGF1 levels and the IGF1 signaling pathway in risk of breast cancer (3, 9, 23). Variants of the *IGF1* gene, which encodes the IGF1 protein, are associated with increased circulating IGF1 levels (14–16) and increased percent breast density (17, 34, 35). However, studies on its association with breast cancer risk have been less conclusive. Our study observed associations between two *IGF1* SNPs, rs1019731 and rs12821878, and breast cancer risk among European women; however, a study from the Breast and Prostate Cancer Cohort Consortium reported no association with breast cancer risk for these SNPs (23). A study by Verheus et al. evaluated the relationship between *IGF1* variants and mammographic density, and reported that the rs12821878 minor allele may be associated with decreased mammographic density (36), which is congruous with the decrease in risk we observed.

Similarly, the *IGFBP3*, *IGF1R*, *IRS1*, and *PI3KCB* genes have a purported relationship with breast cancer risk due to either their role in IGF1 signaling regulation (15, 18, 27), or association with strong breast cancer risk factors (17, 28). However, our null results with regard to the relationship between variants of the *IGFBP3*, *IGF1R*, *IRS1*, and *PI3KCB* genes and, overall, premenopausal and postmenopausal breast cancer risk in European and East Asian women are in concordance with the findings from analyses in the Breast and Prostate Cancer Cohort Consortium (23, 37) and recent GWAS publications (38, 39).

We observed modification of associations with breast cancer risk by menopausal status for four *IG1* SNPs (rs17727841, rs2288378, rs7136446, and rs7956547) in high LD among East Asian women. Menopause-associated declines in IGF1 levels have been previously reported (40, 41) and may explain our findings, and is corroborated by reported effect modification by menopausal status of associations between breast cancer risk factors, such as age, recent leisure-time physical activity and alcohol consumption, and IGF1 and IGFBP3 levels (42). However, the decrease in risk as a result of these SNPs among postmenopausal East Asian women is incongruent with the literature. A meta-analysis reported that IGF1 levels were approximately 7 ng/mL higher among individuals carrying at least one minor allele at the rs7136446 polymorphism compared to homozygous carriers of the major allele (43). Genotype imputation of this SNP in another study produced similar findings (16). Increased IGF1 levels would presumably lead to increased risk of breast cancer; however, our study found the opposite direction of effect.

Analyses by breast tumor subtype revealed potential heterogeneity for rs2288378, representing the four high-LD *IGF1* SNPs, decreasing risk of only ER+ and/or PgR+/HER2− tumors by approximately 45% among East Asian women. One study suggests that positive associations between IGF1 concentrations and breast cancer risk may be restricted to ER+ tumors (9), which may explain why stronger associations were observed for ER+ and/or PgR+ tumors in our study. *In vitro* studies have demonstrated that the IGF and ER signaling pathways are positively reinforced by one another, mediating signaling via the other pathway through increasing expression of key signaling pathway proteins, cross-activation, and stimulating transcriptional activation (13, 44, 45). In addition, interactions with HER2 have been suggested, as cross-talk between IGF1R and HER2 has been implicated in resistance against trastuzumab, a monoclonal antibody used in breast cancer treatment to target HER2 receptors (46, 47). This extensive network of interactions between IGF1, ER, and HER2 signaling pathways, and potential heterogeneity of risk by tumor subtype for *IGF1* genes observed in our study suggest greater complexities in the relationship between IGF1 pathway genes and breast cancer etiology.

Limitations of our study include limited statistical power in stratified and subgroup analyses. Specifically, with the number of cases and controls in our European subgroup and after accounting for multiple comparisons, we were powered to observe effect sizes as low as OR = 1.34 (or OR = 0.75 if protective) for SNPs with MAFs of 0.50, and OR = 1.80 (or OR = 0.56 if protective) for SNPS with MAFs of 0.05. The small sample sizes in the analyses of East Asian women and of tumor subtypes warrant caution in the interpretations, and further large studies are needed to confirm these findings. Differences in demographic, lifestyle, and reproductive factors between cases and controls of the study may be partly attributed to the recruitment methods used in BC where cases were selected using a population-based cancer registry and controls were recruited from a breast mammography program. If those who elect to participate in screening programs are different than those who do not, there is potential for selection bias to be introduced. However, since the study recruitment strategies are unlikely to be related to individuals’ genetic variants in IGF pathway genes, concerns for selection bias are minimized.

In conclusion, our study observed associations between some *IGF1* SNPs and breast cancer risk among European and East Asian women, although evidence of an association for other IGF pathway SNPs was limited. Potential modification of these relationships by menopausal status and breast tumor subtype may warrant further consideration when assessing molecular signaling pathway interactions in the genetic etiology of breast cancer.

## Author Contributions

JS was responsible for the analysis and drafting the manuscript. Drs. KA and JJS are the co-principal investigators of the Canadian Breast Cancer Study (CBCS). Drs. AG, IB, CL, SS, AB-W, and HR are investigators of the CBCS. All co-PIs and investigators of the CBCS were involved in designing and conducting the original study. AL is the Research Project Coordinator for the CBCS and was involved in study planning. LK assisted in the analysis of the physical activity-related variables, and Dr. JMS was responsible for the quality control for the genotype data. Dr. HR also played a supervisory role in the preparation of this manuscript. All authors reviewed and contributed to the submitted version of the paper.

## Conflict of Interest Statement

The authors declare that the research was conducted in the absence of any commercial or financial relationships that could be construed as a potential conflict of interest.
