# Supplementary material for: Polymorphisms of Insulin-Like Growth Factor 1 Pathway Genes and Breast Cancer Risk
Source: Front Oncol. 2016 Jun 8;6:136. doi: 10.3389/fonc.2016.00136 (PMC4896919; doi:10.3389/fonc.2016.00136)
Supplement: Supplementary file 1 [file Table_1.DOCX]

Supplementary Material

Polymorphisms of insulin-like growth factor 1 pathway genes and breast cancer risk

Joy Shi, Kristan J. Aronson1, Anne Grundy, Lindsay C. Kobayashi, Igor Burstyn, Johanna M. Schuetz, Caroline A. Lohrisch, Sandip K. SenGupta, Agnes S. Lai, Angela Brooks-Wilson, John J. Spinelli, Harriet Richardson*

*** Correspondence:** Harriet Richardson: hrichardson@ctg.queensu.ca

**Supplementary Table 1.** Genotype frequencies for SNPs in insulin-like growth factor signaling genes among European and East Asian women

| **Gene** | **SNP** | **Genotype** | **n (%)** | | | | | | | |
| --- | --- | --- | --- | --- | --- | --- | --- | --- | --- | --- |
|  |  |  | **European** | | | | **East Asian** | | | |
|  |  |  | **Case**  **(n = 641)** | | **Controls**  **(n = 806)** | | **Cases**  **(n = 305)** | | **Controls**  **(n = 168)** | |
| *IGF1* | rs6214 | GG | 220 | (34.3) | 261 | (32.4) | 62 | (20.3) | 41 | (24.4) |
|  |  | AG | 323 | (50.4) | 399 | (49.5) | 160 | (52.5) | 90 | (53.6) |
|  |  | AA | 98 | (15.3) | 146 | (18.1) | 83 | (27.2) | 37 | (22.0) |
| *IGF1* | rs1549593 | CC | 487 | (76.0) | 567 | (70.4) | 297 | (97.4) | 162 | (96.4) |
|  |  | AC | 145 | (22.6) | 222 | (27.5) | 8 | (2.6) | 6 | (3.6) |
|  |  | AA | 8 | (1.3) | 16 | (2.0) | 0 | (0.0) | 0 | (0.0) |
| *IGF1* | rs17727841 | CC | 419 | (65.4) | 527 | (65.4) | 227 | (74.4) | 112 | (66.7) |
|  |  | CG | 204 | (31.8) | 255 | (31.6) | 70 | (23.0) | 49 | (29.2) |
|  |  | GG | 18 | (2.8) | 24 | (3.0) | 8 | (2.6) | 7 | (4.2) |
| *IGF1* | rs2288378 | GG | 339 | (52.9) | 440 | (54.6) | 227 | (74.4) | 111 | (66.1) |
|  |  | AG | 265 | (41.3) | 322 | (40.0) | 70 | (23.0) | 50 | (29.8) |
|  |  | AA | 37 | (5.8) | 44 | (5.5) | 8 | (2.6) | 7 | (4.2) |
| *IGF1* | rs7136446 | AA | 196 | (30.6) | 259 | (32.1) | 225 | (73.8) | 110 | (65.5) |
|  |  | AG | 323 | (50.4) | 412 | (51.1) | 72 | (23.6) | 51 | (30.4) |
|  |  | GG | 122 | (19.0) | 135 | (16.8) | 8 | (2.6) | 7 | (4.2) |
| *IGF1* | rs2195239 | GG | 349 | (54.5) | 453 | (56.2) | 95 | (31.2) | 45 | (26.8) |
|  |  | CG | 267 | (41.7) | 301 | (37.3) | 149 | (48.9) | 92 | (54.8) |
|  |  | CC | 25 | (3.9) | 52 | (6.5) | 61 | (20.0) | 31 | (18.5) |
|  |  |  |  |  |  |  |  |  |  |  |
| *IGF1* | rs7956547 | AA | 337 | (52.6) | 429 | (53.2) | 228 | (74.8) | 112 | (66.7) |
|  |  | AG | 269 | (42.0) | 317 | (39.3) | 70 | (23.0) | 50 | (29.8) |
|  |  | GG | 35 | (5.5) | 60 | (7.4) | 7 | (2.3) | 6 | (3.6) |
| *IGF1* | rs1019731 | CC | 509 | (79.4) | 578 | (71.7) | 304 | (99.7) | 168 | (100.0) |
|  |  | AC | 125 | (19.5) | 210 | (26.1) | 1 | (0.3) | 0 | (0.0) |
|  |  | AA | 7 | (1.1) | 18 | (2.2) | 0 | (0.0) | 0 | (0.0) |
| *IGF1* | rs12821878 | GG | 421 | (65.7) | 461 | (57.2) | 278 | (91.2) | 157 | (93.5) |
|  |  | AG | 195 | (30.4) | 301 | (37.3) | 27 | (8.9) | 10 | (6.0) |
|  |  | AA | 25 | (3.9) | 44 | (5.5) | 0 | (0.0) | 1 | (0.6) |
| *IGFBP3* | rs6670 | AA | 389 | (60.7) | 480 | (59.6) | 288 | (94.4) | 164 | (97.6) |
|  |  | AT | 221 | (34.5) | 285 | (35.4) | 16 | (5.3) | 4 | (2.4) |
|  |  | TT | 30 | (4.7) | 41 | (5.1) | 1 | (0.3) | 0 | (0.0) |
| *IGFBP3* | rs2453839 | AA | 415 | (64.7) | 537 | (66.6) | 195 | (63.9) | 100 | (59.5) |
|  |  | AG | 202 | (31.5) | 235 | (29.2) | 97 | (31.8) | 57 | (33.9) |
|  |  | GG | 24 | (3.7) | 34 | (4.2) | 13 | (4.3) | 11 | (6.6) |
| *IGFBP3* | rs3110697 | GG | 219 | (34.2) | 274 | (34.0) | 171 | (56.1) | 85 | (50.6) |
|  |  | AG | 318 | (49.6) | 378 | (46.9) | 112 | (36.7) | 69 | (41.1) |
|  |  | AA | 104 | (16.2) | 154 | (19.1) | 22 | (7.2) | 14 | (8.3) |
| *IGFBP3* | rs2471551 | GG | 408 | (63.7) | 495 | (61.4) | 283 | (92.8) | 161 | (95.8) |
|  |  | CG | 210 | (32.8) | 272 | (33.8) | 22 | (7.2) | 7 | (4.2) |
|  |  | CC | 23 | (3.6) | 39 | (4.8) | 0 | (0.0) | 0 | (0.0) |
| *IGFBP3* | rs2132572 | GG | 393 | (61.3) | 508 | (63.0) | 190 | (62.3) | 95 | (56.6) |
|  |  | AG | 219 | (34.2) | 256 | (31.8) | 99 | (32.5) | 62 | (36.9) |
|  |  | AA | 26 | (4.1) | 41 | (5.1) | 14 | (4.6) | 11 | (6.6) |
|  |  |  |  |  |  |  |  |  |  |  |
| *IGF1R* | rs951715 | AA | 256 | (39.9) | 347 | (43.1) | 90 | (29.5) | 41 | (24.4) |
|  |  | AG | 289 | (45.1) | 363 | (45.0) | 143 | (46.9) | 86 | (51.2) |
|  |  | GG | 96 | (15.0) | 96 | (11.9) | 72 | (23.6) | 41 | (24.4) |
| *IGF1R* | rs2229765 | GG | 208 | (32.5) | 264 | (32.8) | 138 | (45.3) | 73 | (43.5) |
|  |  | AG | 294 | (45.9) | 379 | (47.0) | 134 | (43.9) | 79 | (47.0) |
|  |  | AA | 139 | (21.7) | 163 | (20.2) | 33 | (10.8) | 16 | (9.5) |
| *IGF1R* | rs8038415 | AA | 175 | (27.3) | 209 | (25.9) | 73 | (23.9) | 47 | (28.0) |
|  |  | AG | 304 | (47.4) | 402 | (49.9) | 154 | (50.5) | 86 | (51.2) |
|  |  | GG | 162 | (25.3) | 195 | (24.2) | 78 | (25.6) | 34 | (20.2) |
| *IRS1* | rs1801278 | GG | 554 | (86.4) | 708 | (87.8) | 299 | (98.0) | 160 | (95.2) |
|  |  | AG | 83 | (13.0) | 95 | (11.8) | 6 | (2.0) | 8 | (4.8) |
|  |  | AA | 4 | (0.6) | 2 | (0.3) | 0 | (0.0) | 0 | (0.0) |
| *PI3KCB* | rs12493155 | GG | 181 | (28.2) | 248 | (30.8) | 93 | (30.5) | 50 | (29.8) |
|  |  | AG | 315 | (49.1) | 384 | (47.6) | 152 | (49.8) | 75 | (44.6) |
|  |  | AA | 145 | (22.6) | 174 | (21.6) | 60 | (19.7) | 43 | (25.6) |
| *PI3KCB* | rs524164 | GG | 203 | (31.7) | 235 | (29.2) | 296 | (97.1) | 161 | (95.8) |
|  |  | AG | 308 | (48.1) | 383 | (47.5) | 9 | (3.0) | 7 | (4.2) |
|  |  | AA | 130 | (20.3) | 188 | (23.3) | 0 | (0.0) | 0 | (0.0) |
| *PI3KCB* | rs10513055 | AA | 403 | (62.9) | 482 | (59.8) | 304 | (99.7) | 167 | (99.4) |
|  |  | AC | 217 | (33.9) | 276 | (34.2) | 1 | (0.3) | 1 | (0.6) |
|  |  | CC | 21 | (3.3) | 48 | (6.0) | 0 | (0.0) | 0 | (0.0) |
| *PI3KCB* | rs361072 | AA | 205 | (32.0) | 236 | (29.3) | 296 | (97.1) | 161 | (95.8) |
|  |  | AG | 306 | (47.7) | 385 | (47.8) | 9 | (3.0) | 7 | (4.2) |
|  |  | GG | 130 | (20.3) | 185 | (23.0) | 0 | (0.0) | 0 | (0.0) |
