# Supplementary material for: Polymorphisms of Insulin-Like Growth Factor 1 Pathway Genes and Breast Cancer Risk
Source: Front Oncol. 2016 Jun 8;6:136. doi: 10.3389/fonc.2016.00136 (PMC4896919; doi:10.3389/fonc.2016.00136)
Supplement: Supplementary file 2 [file Table_2.DOCX]

Supplementary Material

Polymorphisms of insulin-like growth factor 1 pathway genes and breast cancer risk

Joy Shi, Kristan J. Aronson1, Anne Grundy, Lindsay C. Kobayashi, Igor Burstyn, Johanna M. Schuetz, Caroline A. Lohrisch, Sandip K. SenGupta, Agnes S. Lai, Angela Brooks-Wilson, John J. Spinelli, Harriet Richardson*

*** Correspondence:** Harriet Richardson: hrichardson@ctg.queensu.ca

**Supplementary Table 2.** Genotype frequencies for SNPs in insulin-like growth factor signaling genes among premenopausal and postmenopausal European and East Asian women

| **Gene** | **SNP** | **Genotype** | **n (%)** | | | | | | | | | | | | | | | |
| --- | --- | --- | --- | --- | --- | --- | --- | --- | --- | --- | --- | --- | --- | --- | --- | --- | --- | --- |
|  |  |  | **European** | | | | | | | | **East Asian** | | | | | | | |
|  |  |  | **Premenopausal** | | | | **Postmenopausal** | | | | **Premenopausal** | | | | **Postmenopausal** | | | |
|  |  |  | **Case**  **(n = 189)** | | **Controls**  **(n = 275)** | | **Cases**  **(n = 451)** | | **Controls**  **(n = 531)** | | **Case**  **(n = 127)** | | **Controls**  **(n = 79)** | | **Cases**  **(n = 178)** | | **Controls**  **(n = 89)** | |
| *IGF1* | rs6214 | GG | 70 | (37.0) | 94 | (34.2) | 150 | (33.3) | 167 | (31.5) | 32 | (25.2) | 20 | (25.3) | 30 | (16.9) | 21 | (23.6) |
|  |  | AG | 83 | (43.9) | 138 | (50.2) | 240 | (53.2) | 261 | (49.2) | 65 | (51.2) | 39 | (49.4) | 95 | (53.4) | 51 | (57.3) |
|  |  | AA | 36 | (19.1) | 43 | (15.6) | 61 | (13.5) | 103 | (19.4) | 30 | (23.6) | 20 | (25.3) | 53 | (29.8) | 17 | (19.1) |
| *IGF1* | rs1549593 | CC | 138 | (73.0) | 196 | (71.3) | 348 | (77.2) | 371 | (69.9) | 123 | (96.9) | 76 | (96.2) | 174 | (97.8) | 86 | (96.6) |
|  |  | AC | 48 | (25.4) | 76 | (27.6) | 97 | (21.5) | 146 | (27.5) | 4 | (3.2) | 3 | (3.8) | 4 | (2.3) | 3 | (3.4) |
|  |  | AA | 3 | (1.6) | 3 | (1.1) | 5 | (1.1) | 13 | (2.5) | 0 | (0.0) | 0 | (0.0) | 0 | (0.0) | 0 | (0.0) |
| *IGF1* | rs17727841 | CC | 132 | (69.8) | 186 | (67.6) | 286 | (63.4) | 341 | (64.2) | 86 | (67.7) | 60 | (76.0) | 141 | (79.2) | 52 | (58.4) |
|  |  | CG | 53 | (28.0) | 87 | (31.6) | 151 | (33.5) | 168 | (31.6) | 37 | (29.1) | 16 | (20.3) | 33 | (18.5) | 33 | (37.1) |
|  |  | GG | 4 | (2.1) | 2 | (0.7) | 14 | (3.1) | 22 | (4.1) | 4 | (3.2) | 3 | (3.8) | 4 | (2.3) | 4 | (4.5) |
| *IGF1* | rs2288378 | GG | 111 | (58.7) | 149 | (54.2) | 227 | (50.3) | 291 | (54.8) | 86 | (67.7) | 60 | (76.0) | 141 | (79.2) | 51 | (57.3) |
|  |  | AG | 70 | (37.0) | 118 | (42.9) | 195 | (43.2) | 204 | (38.4) | 37 | (29.1) | 16 | (20.3) | 33 | (18.5) | 34 | (38.2) |
|  |  | AA | 8 | (4.2) | 8 | (2.9) | 29 | (6.4) | 36 | (6.8) | 4 | (3.2) | 3 | (3.8) | 4 | (2.3) | 4 | (4.5) |
| *IGF1* | rs7136446 | AA | 67 | (35.5) | 89 | (32.4) | 129 | (28.6) | 170 | (32.0) | 86 | (67.7) | 59 | (74.7) | 139 | (78.1) | 51 | (57.3) |
|  |  | AG | 94 | (49.7) | 141 | (51.3) | 229 | (50.8) | 271 | (51.0) | 37 | (29.1) | 17 | (21.5) | 35 | (19.7) | 34 | (38.2) |
|  |  | GG | 28 | (14.8) | 45 | (16.4) | 93 | (20.6) | 90 | (17.0) | 4 | (3.2) | 3 | (3.8) | 4 | (2.3) | 4 | (4.5) |
| *IGF1* | rs2195239 | GG | 106 | (56.1) | 162 | (58.9) | 242 | (53.7) | 291 | (54.8) | 41 | (32.3) | 22 | (27.9) | 54 | (30.3) | 23 | (25.8) |
|  |  | CG | 77 | (40.7) | 102 | (37.1) | 190 | (42.1) | 199 | (37.5) | 64 | (50.4) | 47 | (59.5) | 85 | (47.8) | 45 | (50.6) |
|  |  | CC | 6 | (3.2) | 11 | (4.0) | 19 | (4.2) | 41 | (7.7) | 22 | (17.3) | 10 | (12.7) | 39 | (21.9) | 21 | (23.6) |
| *IGF1* | rs7956547 | AA | 108 | (57.1) | 151 | (54.9) | 228 | (50.6) | 278 | (52.4) | 87 | (68.5) | 60 | (76.0) | 141 | (79.2) | 52 | (58.4) |
|  |  | AG | 71 | (37.6) | 112 | (40.7) | 198 | (43.9) | 205 | (38.6) | 37 | (29.1) | 16 | (20.3) | 33 | (18.5) | 34 | (38.2) |
|  |  | GG | 10 | (5.3) | 12 | (4.4) | 25 | (5.5) | 48 | (9.0) | 3 | (2.4) | 3 | (3.8) | 4 | (2.3) | 3 | (3.4) |
| *IGF1* | rs1019731 | CC | 150 | (79.4) | 197 | (71.6) | 358 | (79.4) | 381 | (71.8) | 127 | (100.0) | 79 | (100.0) | 177 | (99.4) | 89 | (100.0) |
|  |  | AC | 38 | (20.1) | 74 | (26.9) | 87 | (19.3) | 136 | (25.6) | 0 | (0.0) | 0 | (0.0) | 1 | (0.6) | 0 | (0.0) |
|  |  | AA | 1 | (0.5) | 4 | (1.5) | 6 | (1.3) | 14 | (2.6) | 0 | (0.0) | 0 | (0.0) | 0 | (0.0) | 0 | (0.0) |
| *IGF1* | rs12821878 | GG | 127 | (67.2) | 155 | (56.4) | 293 | (65.0) | 306 | (57.6) | 293 | (90.6) | 74 | (93.7) | 163 | (91.6) | 83 | (93.3) |
|  |  | AG | 56 | (29.6) | 103 | (37.5) | 139 | (30.8) | 198 | (37.3) | 139 | (9.5) | 4 | (5.1) | 15 | (8.4) | 6 | (6.7) |
|  |  | AA | 6 | (3.2) | 17 | (6.2) | 19 | (4.2) | 27 | (5.1) | 19 | (0.0) | 1 | (1.3) | 0 | (0.0) | 0 | (0.0) |
| *IGFBP3* | rs6670 | AA | 119 | (63.0) | 157 | (57.1) | 270 | (59.9) | 323 | (60.8) | 121 | (95.3) | 79 | (100.0) | 167 | (93.8) | 85 | (95.5) |
|  |  | AT | 61 | (32.3) | 104 | (37.8) | 159 | (35.3) | 181 | (34.1) | 5 | (3.9) | 0 | (0.0) | 11 | (6.2) | 4 | (4.5) |
|  |  | TT | 8 | (4.2) | 14 | (5.1) | 22 | (4.9) | 27 | (5.1) | 1 | (0.8) | 0 | (0.0) | 0 | (0.0) | 0 | (0.0) |
| *IGFBP3* | rs2453839 | AA | 119 | (63.0) | 182 | (66.2) | 295 | (65.4) | 355 | (66.9) | 78 | (61.4) | 48 | (60.8) | 117 | (65.7) | 52 | (58.4) |
|  |  | AG | 66 | (34.9) | 76 | (27.6) | 136 | (30.2) | 159 | (29.9) | 42 | (33.1) | 22 | (27.9) | 55 | (30.9) | 35 | (39.3) |
|  |  | GG | 4 | (2.1) | 17 | (6.2) | 20 | (4.4) | 17 | (3.2) | 7 | (5.5) | 9 | (11.4) | 6 | (3.4) | 2 | (2.3) |
| *IGFBP3* | rs3110697 | GG | 64 | (33.9) | 95 | (34.6) | 154 | (34.2) | 179 | (33.7) | 66 | (52.0) | 42 | (53.2) | 105 | (59.0) | 43 | (48.3) |
|  |  | AG | 99 | (52.4) | 137 | (49.8) | 219 | (48.6) | 241 | (45.4) | 52 | (40.9) | 28 | (35.4) | 60 | (33.7) | 41 | (46.1) |
|  |  | AA | 26 | (13.8) | 43 | (15.6) | 78 | (17.3) | 111 | (20.9) | 9 | (7.1) | 9 | (11.4) | 13 | (7.3) | 5 | (5.6) |
| *IGFBP3* | rs2471551 | GG | 125 | (66.1) | 171 | (62.2) | 282 | (62.5) | 324 | (61.0) | 116 | (91.3) | 78 | (98.7) | 167 | (93.8) | 83 | (93.3) |
|  |  | CG | 57 | (30.2) | 88 | (32.0) | 153 | (33.9) | 184 | (34.7) | 11 | (8.7) | 1 | (1.3) | 11 | (6.2) | 6 | (6.7) |
|  |  | CC | 7 | (3.7) | 16 | (5.8) | 16 | (3.6) | 23 | (4.3) | 0 | (0.0) | 0 | (0.0) | 0 | (0.0) | 0 | (0.0) |
| *IGFBP3* | rs2132572 | GG | 117 | (61.9) | 178 | (64.7) | 275 | (61.0) | 330 | (62.2) | 75 | (59.1) | 42 | (53.2) | 115 | (64.6) | 53 | (59.6) |
|  |  | AG | 63 | (33.3) | 90 | (32.7) | 156 | (34.6) | 166 | (31.3) | 44 | (34.7) | 29 | (36.7) | 55 | (30.9) | 33 | (37.1) |
|  |  | AA | 8 | (4.2) | 7 | (2.6) | 18 | (4.0) | 34 | (6.4) | 8 | (6.3) | 8 | (10.1) | 6 | (3.4) | 3 | (3.4) |
| *IGF1R* | rs951715 | AA | 78 | (41.3) | 130 | (47.3) | 178 | (39.5) | 217 | (40.9) | 39 | (30.7) | 20 | (25.3) | 51 | (28.7) | 21 | (23.6) |
|  |  | AG | 75 | (39.7) | 115 | (41.8) | 214 | (47.5) | 248 | (46.7) | 56 | (44.1) | 35 | (44.3) | 87 | (48.9) | 51 | (57.3) |
|  |  | GG | 36 | (19.1) | 30 | (10.9) | 59 | (13.1) | 66 | (12.4) | 32 | (25.2) | 24 | (30.4) | 40 | (22.5) | 17 | (19.1) |
| *IGF1R* | rs2229765 | GG | 53 | (28.0) | 91 | (33.1) | 154 | (34.2) | 173 | (32.6) | 58 | (45.7) | 28 | (35.4) | 80 | (44.9) | 45 | (50.6) |
|  |  | AG | 98 | (51.9) | 129 | (46.9) | 196 | (43.5) | 250 | (47.1) | 52 | (40.9) | 41 | (51.9) | 82 | (46.1) | 38 | (42.7) |
|  |  | AA | 38 | (20.1) | 55 | (20.0) | 101 | (22.4) | 108 | (20.3) | 17 | (13.4) | 10 | (12.7) | 16 | (9.0) | 6 | (6.7) |
| *IGF1R* | rs8038415 | AA | 49 | (25.9) | 70 | (25.5) | 125 | (27.7) | 139 | (26.2) | 33 | (26.0) | 18 | (22.8) | 40 | (22.5) | 29 | (32.6) |
|  |  | AG | 84 | (44.4) | 132 | (48.0) | 220 | (48.8) | 270 | (50.9) | 58 | (45.7) | 41 | (51.9) | 96 | (53.9) | 45 | (50.6) |
|  |  | GG | 56 | (29.6) | 73 | (26.6) | 106 | (23.5) | 122 | (23.0) | 36 | (28.4) | 20 | (25.3) | 42 | (23.6) | 14 | (15.7) |
| *IRS1* | rs1801278 | GG | 167 | (88.4) | 233 | (84.7) | 386 | (85.6) | 475 | (89.5) | 126 | (99.2) | 77 | (97.5) | 173 | (97.2) | 83 | (93.3) |
|  |  | AG | 21 | (11.1) | 41 | (14.9) | 62 | (13.8) | 54 | (10.2) | 1 | (0.8) | 2 | (2.5) | 5 | (2.8) | 6 | (6.7) |
|  |  | AA | 1 | (0.5) | 1 | (0.4) | 3 | (0.7) | 1 | (0.2) | 0 | (0.0) | 0 | (0.0) | 0 | (0.0) | 0 | (0.0) |
| *PI3KCB* | rs12493155 | GG | 47 | (24.9) | 90 | (32.7) | 134 | (29.7) | 158 | (29.8) | 34 | (26.8) | 24 | (30.4) | 59 | (33.2) | 26 | (29.2) |
|  |  | AG | 102 | (54.0) | 122 | (44.4) | 212 | (47.0) | 262 | (49.3) | 72 | (56.7) | 36 | (45.6) | 80 | (44.9) | 39 | (43.8) |
|  |  | AA | 40 | (21.2) | 63 | (22.9) | 105 | (23.3) | 111 | (20.9) | 21 | (16.5) | 19 | (24.1) | 39 | (21.9) | 24 | (27.0) |
| *PI3KCB* | rs524164 | GG | 62 | (32.8) | 88 | (32.0) | 141 | (31.3) | 147 | (27.7) | 125 | (98.4) | 74 | (93.7) | 171 | (96.1) | 87 | (97.8) |
|  |  | AG | 92 | (48.7) | 119 | (43.3) | 215 | (47.7) | 264 | (49.7) | 2 | (1.6) | 5 | (6.3) | 7 | (3.9) | 2 | (2.3) |
|  |  | AA | 35 | (18.5) | 68 | (24.7) | 95 | (21.1) | 120 | (22.6) | 0 | (0.0) | 0 | (0.0) | 0 | (0.0) | 0 | (0.0) |
| *PI3KCB* | rs10513055 | AA | 118 | (62.4) | 162 | (58.9) | 284 | (63.0) | 320 | (60.3) | 127 | (100.0) | 78 | (98.7) | 177 | (99.4) | 89 | (100.0) |
|  |  | AC | 65 | (34.4) | 96 | (34.9) | 152 | (33.7) | 180 | (33.9) | 0 | (0.0) | 1 | (1.3) | 1 | (0.6) | 0 | (0.0) |
|  |  | CC | 6 | (3.2) | 17 | (6.2) | 15 | (3.3) | 31 | (5.8) | 0 | (0.0) | 0 | (0.0) | 0 | (0.0) | 0 | (0.0) |
| *PI3KCB* | rs361072 | AA | 63 | (33.3) | 89 | (32.4) | 142 | (31.5) | 147 | (27.7) | 125 | (98.4) | 74 | (93.7) | 171 | (96.1) | 87 | (97.8) |
|  |  | AG | 91 | (48.2) | 118 | (42.9) | 214 | (47.5) | 267 | (50.3) | 2 | (1.6) | 5 | (6.3) | 7 | (3.9) | 2 | (2.3) |
|  |  | GG | 35 | (18.5) | 68 | (24.7) | 95 | (21.1) | 117 | (22.0) | 0 | (0.0) | 0 | (0.0) | 0 | (0.0) | 0 | (0.0) |
